# Supplementary material for: Variations in the oral microbiome and metabolome of methamphetamine users
Source: mSystems. 2023 Dec 19;9(1):e00991-23. doi: 10.1128/msystems.00991-23 (PMC10804968; doi:10.1128/msystems.00991-23)
Supplement: Supplemental figures — Fig. S1-S9. [file msystems.00991-23-s0001.docx]

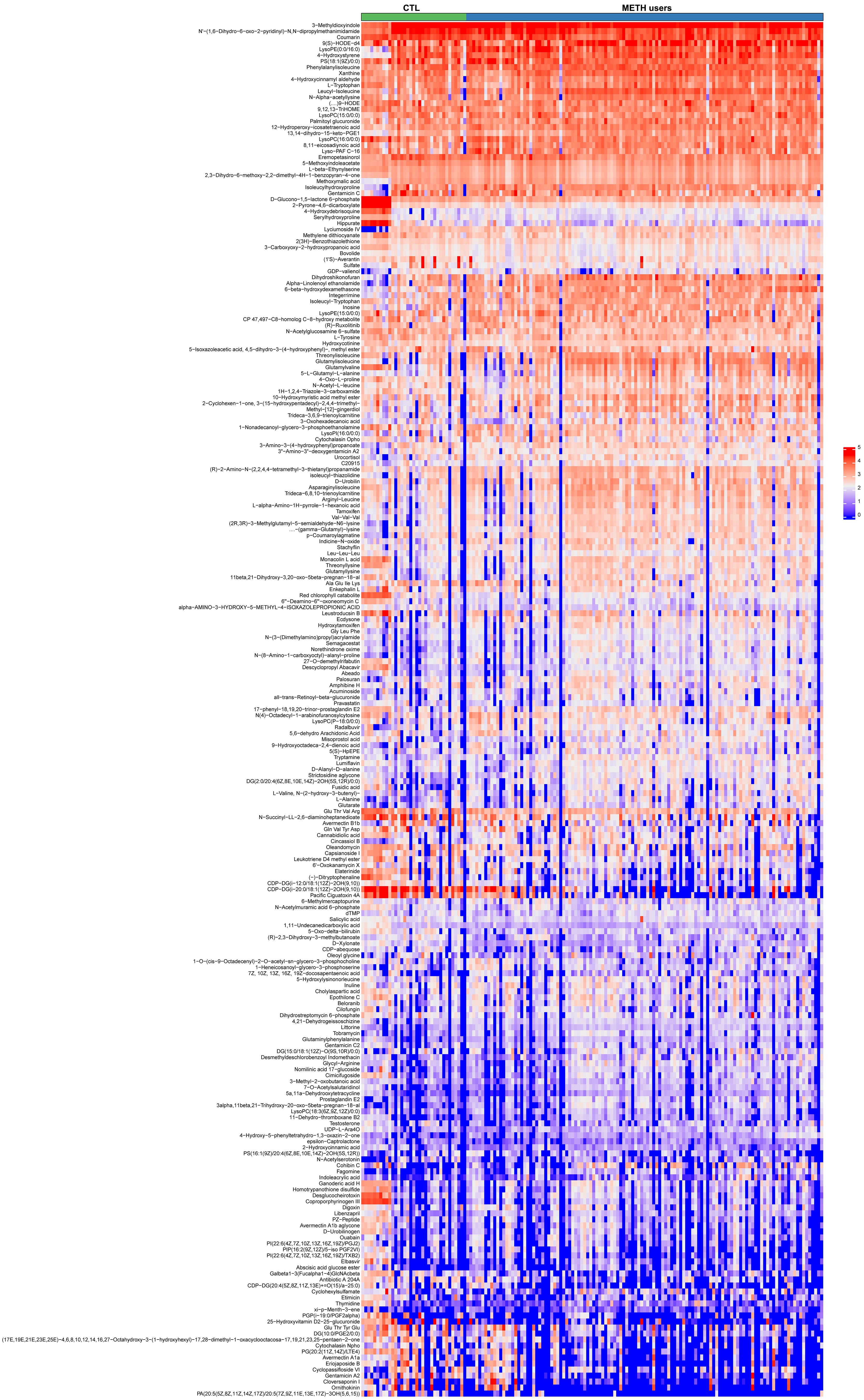


**Supplementary figure 1** Heat map of relative abundance of all metabolites (n = 229). The metabolite heat map was plotted after Log10 conversion (to prevent expressions with a quantification of 0, the value of each quantification result was added by 1).


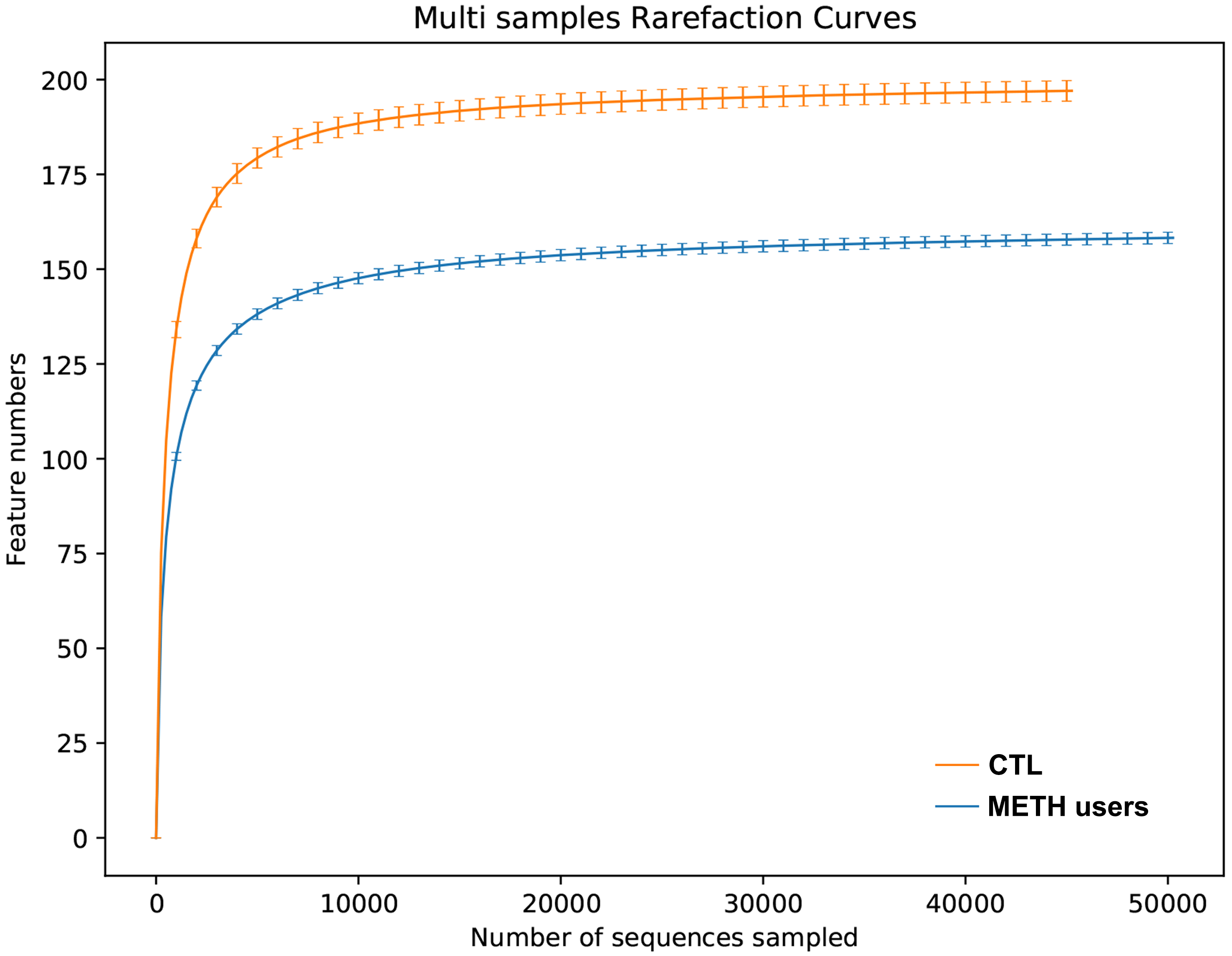


**Supplementary figure 2** The curvilinear relationship between sample size and OTU richness. The OTU richness estimates for all samples are essentially close to saturation.


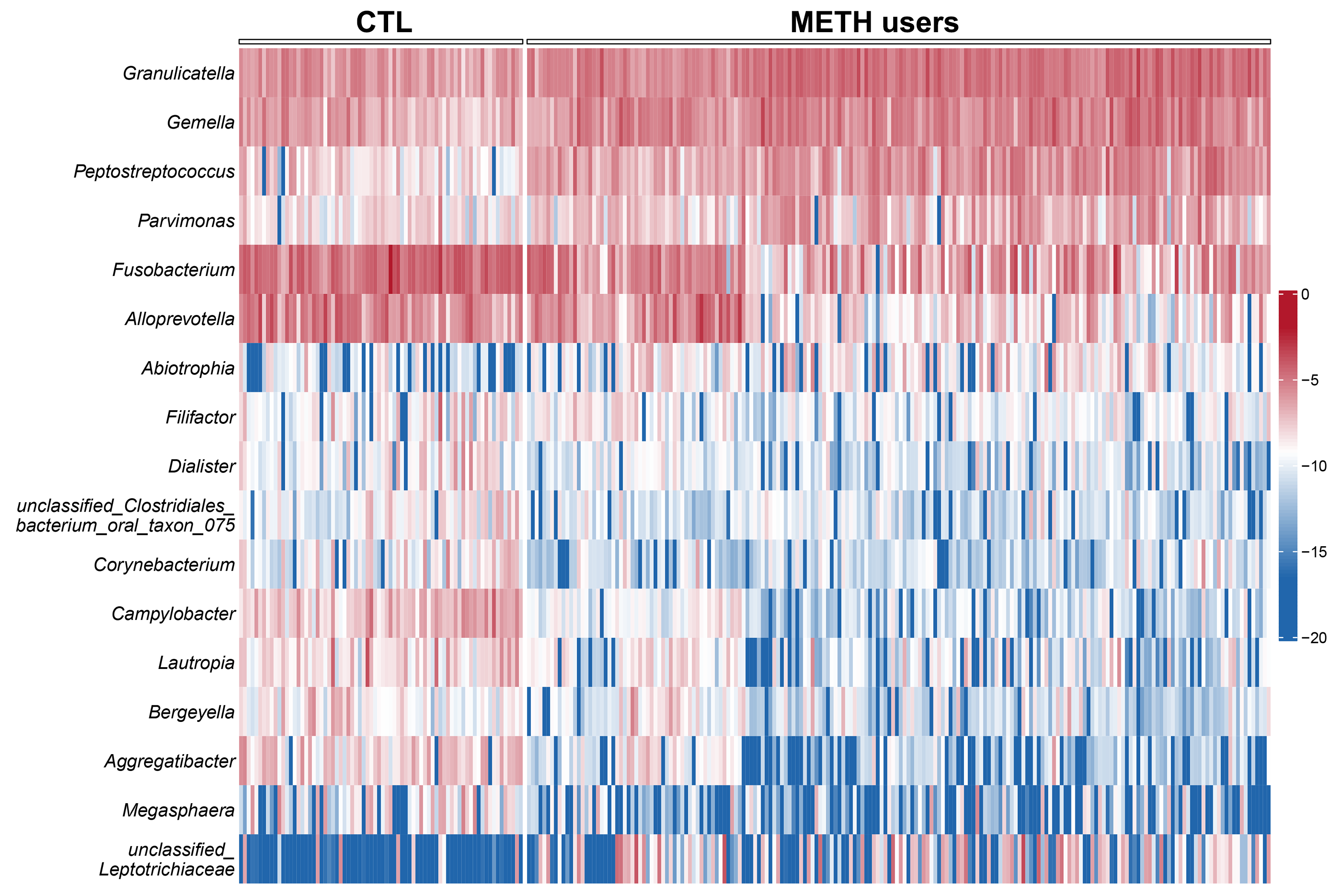


**Supplementary figure 3** Heatmap of the relative abundance of key bacteria (n = 17). The log2 transformed hea map of the microbiota is plotted relatively to a 0 value of 1E-05.


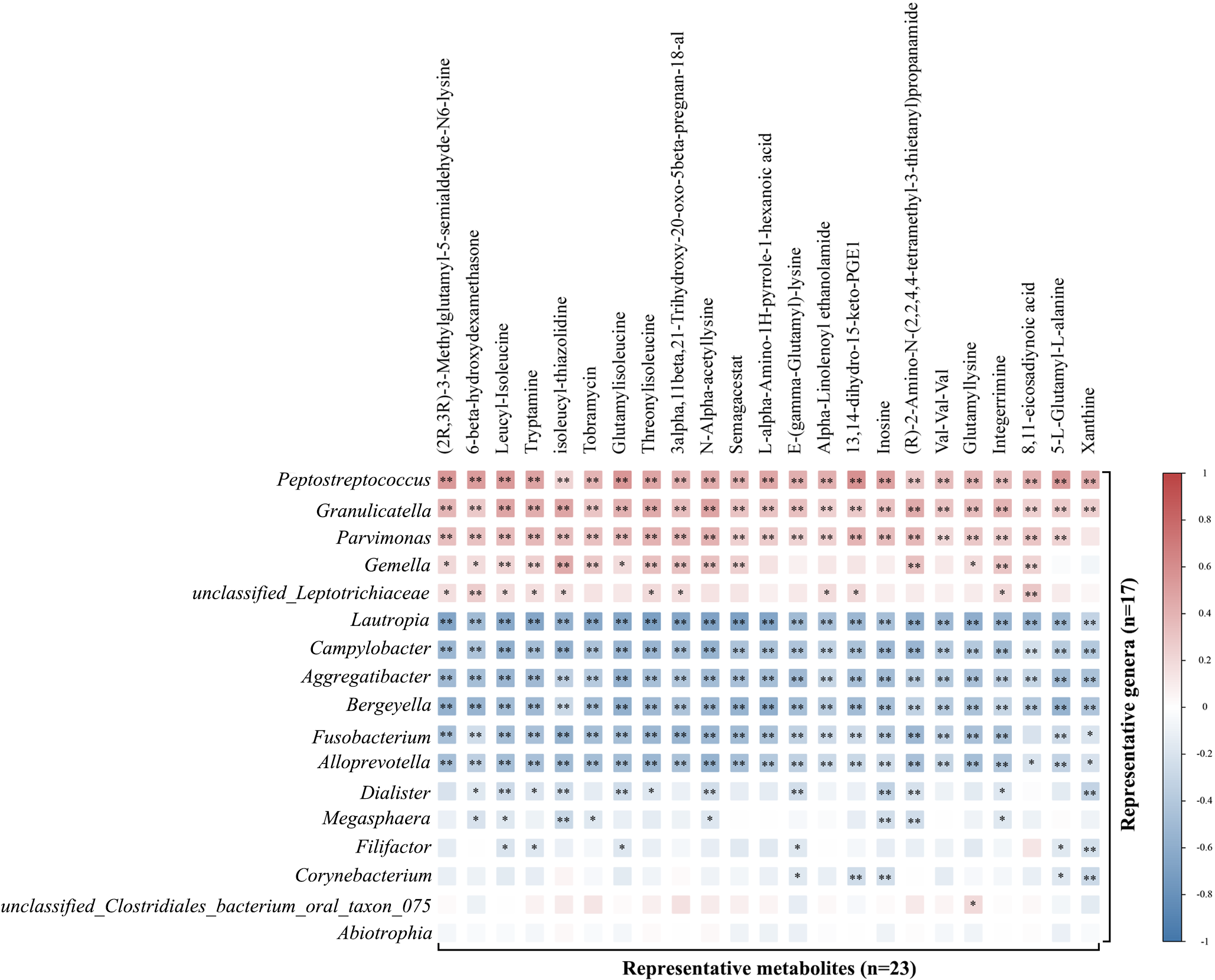


**Supplementary figure 4** Integrated association network of interactions between key microbiota and metabolites in the group of METH users. * P < 0.05, ** P < 0.01.


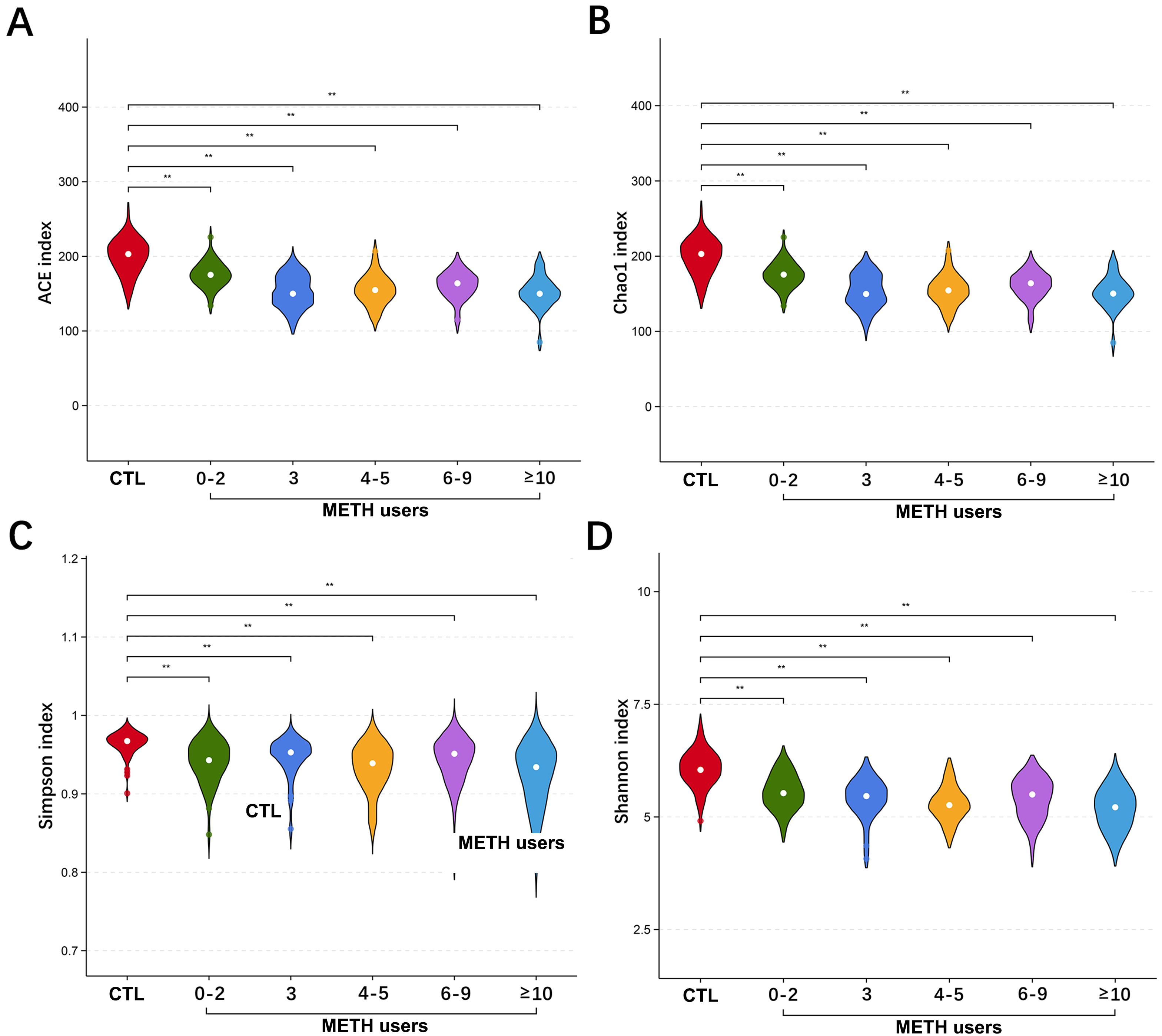


**Supplementary figure 5** Alpha diversity analysis of oral microbiota in six groups of samples. Each experiment is repeated three times. ** P < 0.01.


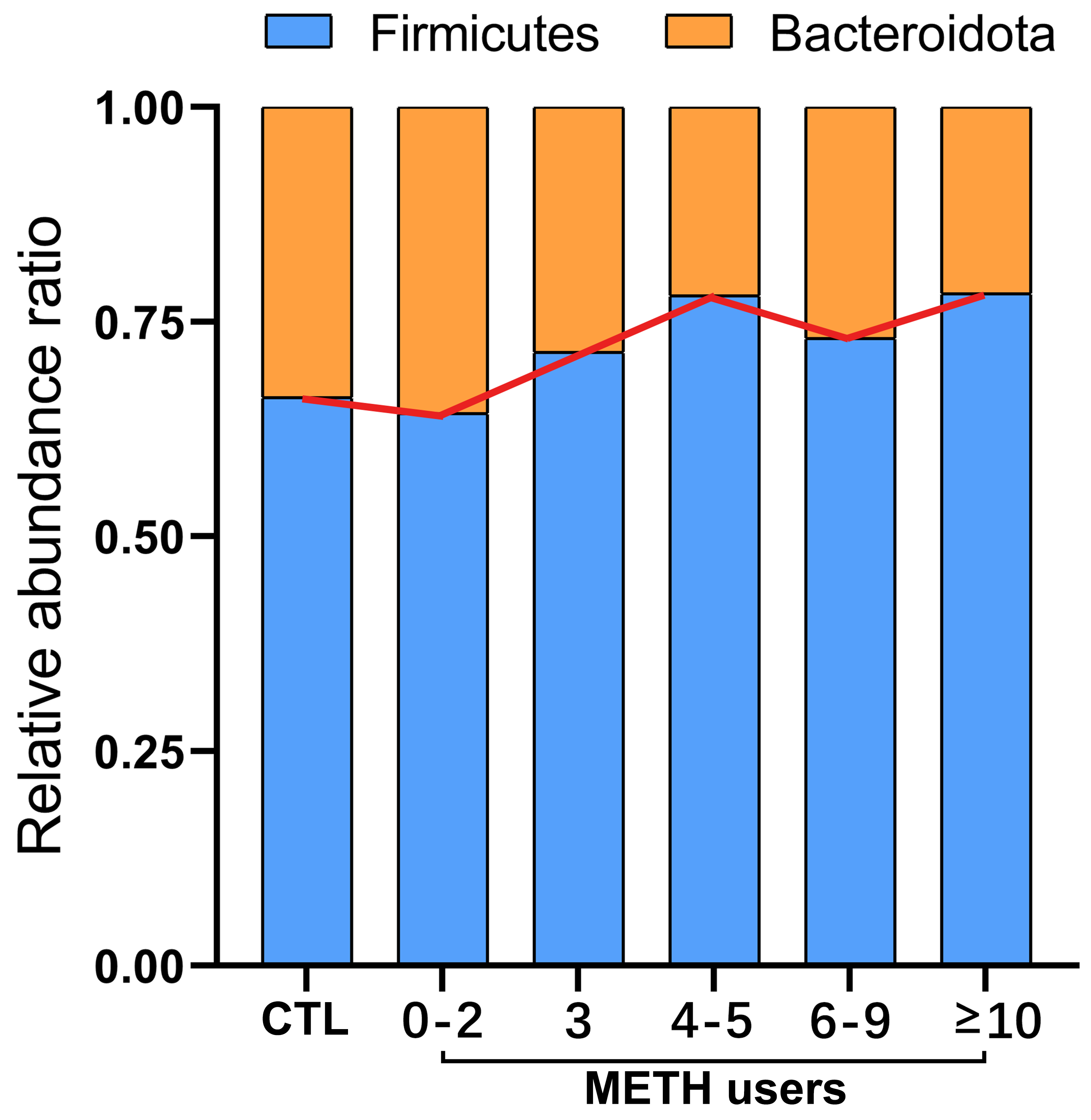


**Supplementary figure 6** The ratio of Firmicutes to Bacteroidota changes in six groups.


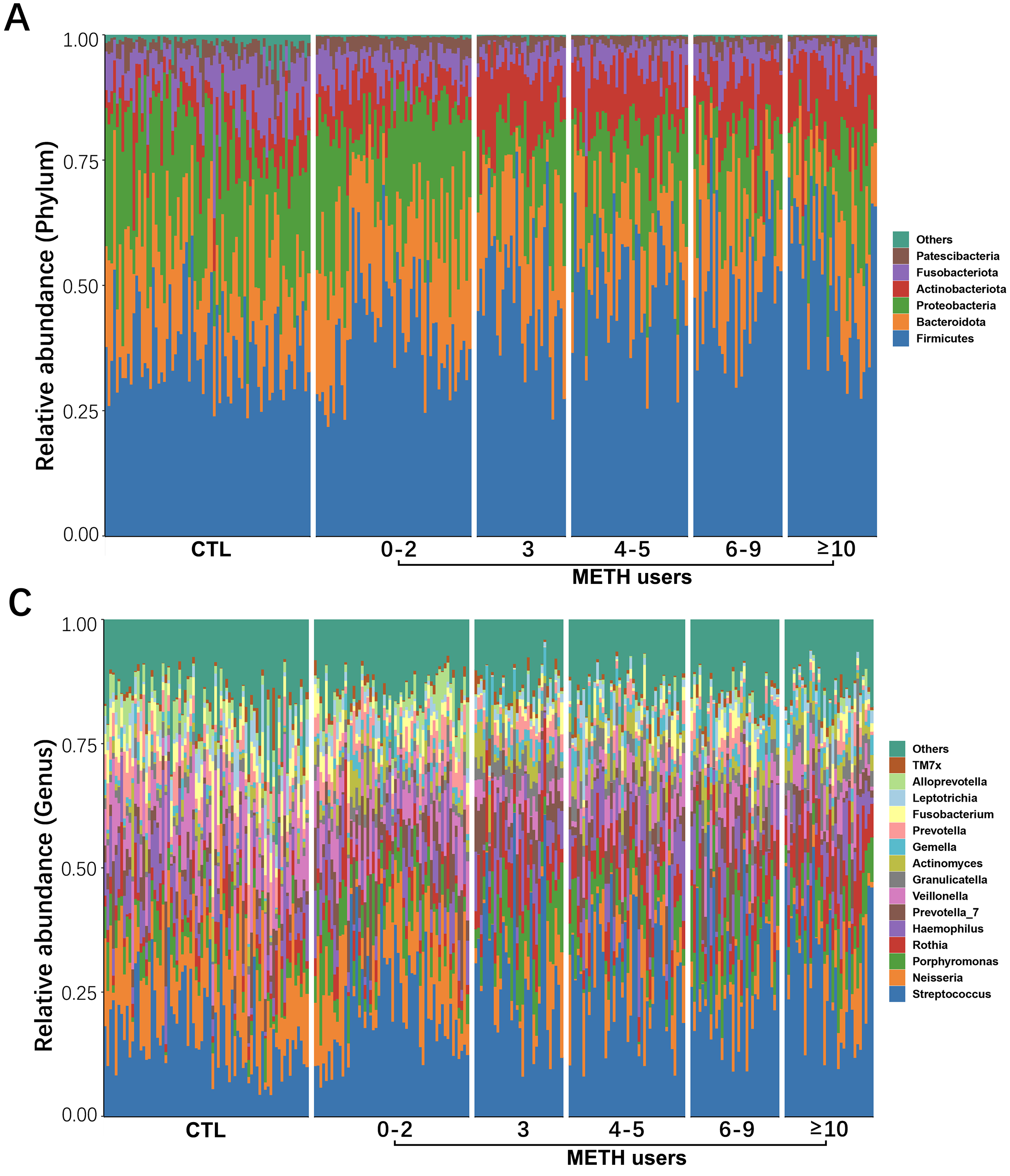


**Supplementary figure 7** Characterization of the oral microbiota in six groups of samples. (A) Compositions of the oral microbiota at the phylum level for all samples. (B) Composition of the oral microbiota at the genus level for all samples.





**Supplementary figure 8** Study design and flow chart based on the duration of drug use. A total of 383 samples are selected for microbial sequencing analysis. The drug users are divided into discovery and validation stages according to their durations of drug use.





**Supplementary figure 9** Study design and flow chart based on geographic regions. A total of 185 samples are selected for microbial sequencing and are divided into a discovery phase and a validation phase based on the geographic regions of the samples.
